# Phytoremediation of industrial mines wastewater using water hyacinth

**DOI:** 10.1080/15226514.2016.1216078

**Published:** 2016-08-23

**Authors:** Priyanka Saha, Omkar Shinde, Supriya Sarkar

**Affiliations:** ^a^ Environment Research Group, RD &T, Tata Steel Ltd, Jamshedpur, India

**Keywords:** Phytoremediation, hexavalent chromium, water hyacinth

## Abstract

The wastewater at Sukinda chromite mines (SCM) area of Orissa (India) showed high levels of toxic hexavalent chromium (Cr VI). Wastewater from chromium-contaminated mines exhibit potential threats for biotic community in the vicinity. The aim of the present investigation is to develop a suitable phytoremediation technology for the effective removal of toxic hexavalent chromium from mines wastewater. A water hyacinth species *Eichhornia crassipes* was chosen to remediate the problem of Cr (VI) pollution from wastewater. It has been observed that this plant was able to remove 99.5% Cr (VI) of the processed water of SCM in 15 days. This aquatic plant not only removed hexavalent Cr, but is also capable of reducing total dissolved solids (TDS), biological oxygen demand (BOD), chemical oxygen demand (COD), and other elements of water also. Large-scale experiment was also performed using 100 L of water from SCM and the same removal efficiency was achieved.

## Introduction

In recent days, groundwater quality is decreasing day by day due to rapid urbanization and fast industrial growth. Discharging of untreated industrial wastewater and domestic household water into water bodies is polluting water quality. Contamination of water in Sukinda (Orissa) chromite mining areas is a serious environmental problem (ATSDR 1998; USEPA 1998; Misra *et al.* 1994). India is the second largest producer of chromite in the world. The state of Orissa in India accounts for 98% of total chromite reserves of the country and the Sukinda chromite mine area of Orissa contributes to about 97% of the total chromite reserve of the state (IBM 2004). Open-cast chromite mining activity causes various environmental problems due to the release of Cr (VI). Chromium exists in two stable states, *i.e.*, hexavalent chromium (Cr VI) and trivalent chromium (Cr III) of which Cr (VI) is the most toxic form. Chromium (III) is essential for breaking down sugar, fat, and protein inside an animal's body, therefore making it essential for good health. In contrast, hexavalent chromium can be harmful to the health of anyone exposed to it over long periods of time. Inhaling or ingesting hexavalent chromium over time can cause nosebleeds, ulcers, convulsions, kidney and liver damage, various cancers, and/or death (ATSDR 1998).

Most of the remedial technologies for the removal of heavy metals are quite expensive and injurious to health. Several conventional methods are available for treatment of chromium from industrial effluents like membrane filtration (Ndiaye *et al.* 2005), precipitation (Pathasarathy *et al.* 1986), nanofiltration (Simons 1993; Religa *et al.* 2010; Abhang *et al.* 2013), ion exchange (Ruixia *et al.* 2002), electrocoagulation flotation (Hu *et al.* 2005), and adsorption (Mohapatra *et al.* 2004). These processes have significant disadvantages, which are, for instance, incomplete removal, high-energy requirements, and production of toxic sludge. Economies of developing countries like India have other investment priority. Therefore, they cannot afford the high price involved in the removal of heavy metals from wastewater. Contrary to this, phytoremediation, *i.e.*, removal of metals through plants offers an eco-friendly and cost-effective methodology. Phytoremediation, which is the use of plants and their associated microorganisms, is one of the recent technologies which guarantee an effective, economical, and sustainable means to achieve this end for developing countries because they are cheaper to make and a little skill is required to operate them. This technique is a cost-effective plant-based approach for removal of heavy metals from water (Terry and Banuelos 2000; Mohanty *et al.* 2005b, Mohanty and Patra 2011; Mohanty 2015). Phytoremediation can be achieved through different methods like phytoextraction (Kumar *et al.* 1995; Tangahu *et al.* 2011), rhizofiltration (Dushenkov *et al.* 1995; Elias *et al.* 2014), phytostabilization (Salt *et al.* 1995), and phytotransformation/phytodegradation (Susarla *et al.* 2002). The success of phytoremediation mainly depends on the photosynthetic activity and the growth rate of plants.

Several species of aquatic macrophytes such as water hyacinth (*Eichhornia* sp.), duckweeds (*Lemna* sp., *Spirodella* sp.), smallwater fern (*Azolla* sp.), and water lettuce (*Pistia* sp.) have been used for the removal of heavy metals from wastewater (Okunowo and Ogunkanmi 2010; Suhag *et al.* 2011; Rai 2008; Gupta *et al.* 2012; Ajibade *et al.* 2013; Saha *et al.* 2015). Water hyacinth, which is the world's most noxious aquatic plant, has a prolific growth rate and thus the potential to remove most of heavy metals including Cr (VI) (Xia and Ma 2005). There are seven species of water hyacinth—the best known being the common water hyacinth, *Eichhornia crassipes*, which is a perennial free floating aquatic plant belonging to the family Pontederiaceae. This plant has high nitrogen content and in combination with cow dung, it can be used for biogas production (Bhattacharya and Pawan 2010). Its enormous biomass production rate, its high tolerance to pollution, and its heavy metal and nutrient absorption capacities (Chanakya *et al.* 1993; Singhal and Rai 2003; Ingole and Bhole 2003; Liao and Chang 2004; Jayaweera and Kasturiarachchi 2004; Swarnalatha *et al.* 2015) qualify it for use in wastewater treatment ponds. For example, studies have shown that about 1 million L/day of domestic sewage could be treated over an area of 1 ha through water hyacinths, reducing the biological oxygen demand (BOD) and chemical oxygen demand (COD) by 89% and 71%, respectively. Because of their relatively high protein content and abundance in tropical and subtropical countries, a significant number of research studies have been carried out to find the potential for the utilization of water hyacinths as a fertilizer.

Under present investigation, the Cr(VI) removal efficiency of water hyacinth from processed water from SCM was evaluated. Chromium, a nonessential micronutrient for normal plant metabolism, has been reported to be one of the most toxic heavy metals present in wastewater discharges from electroplating, dye and pigment manufacturing, wood preserving, and leather tanning industries. Also, Cr is mobile, and has a long residence time in surface water and groundwater. Chromium toxicity affects the plant growth and metabolism, which includes stunted growth, chlorosis, reduced crop yield, delayed germination, senescence, premature leaf fall, biochemical lesions, enzymatic changes, and reduced biosynthesis (Panda and Patra 1997a, b, 1998; Zayed *et al.* 1998a, b; Srivastava *et al.* 1999; Zayed and Terry 2003; Mohanty *et al.* 2005a, 2009, 2010). Excessive Cr accumulation in the plant tissue can be toxic to the plants, affecting several physiological and biochemical processes and growth.

Cr(VI) is a highly toxic form of chromium, which is generated throughout the world due to various industrial activities (viz. chromite mining, Cr plating, chemical manufacturing, etc.) causing contamination of the water bodies within its vicinity. This toxic Cr(VI)-laden water has to be detoxified before its discharge as per the norms laid down by environment regulatory authorities. Many researchers try to find out a suitable method of treating hexavalent chromium for a long run. The conventional method is completely chemical based and residual chemicals can cause a detrimental impact on environment in the long run. Our commitment toward the environment is to develop a novel and sustainable solutions for remediation of toxic hexavalent chromium. Phytoremediation-eco-friendly, cost-effective method helps to achieve statutory norms by regulatory authority in a more eco-friendly way. Saves high chemical expenditures required in conventional method. This cost-effective system can be employed even in remote areas without any basic infrastructure. The aim of the present study was to evaluate the phytoremediation potential of water hyacinth (*Eichhornia crassipes*) to remove Cr (VI) from processed water of SCM.

## Materials and methods

### Plant materials

The study was conducted by using water hyacinth weeds (*Eichhornia crassipes*) which served as tool of phytoremediation. These aquatic weeds were used for reduction of Cr^+6^ from contaminated untreated mine wastewater. Plants were collected from local area of Jamshedpur, India. After collection, they were rinsed with tap water to remove any epiphytes and insect larvae grown on plants. A 1-week acclimatization period was set to stabilize the plant. The plants were placed in tub with tap water without addition of any nutrient media under natural sunlight for 1 week to let them adapt to the new environment, and then the plants of the same size were selected for the following experiments.

### Sampling of water

**Figure 1.**
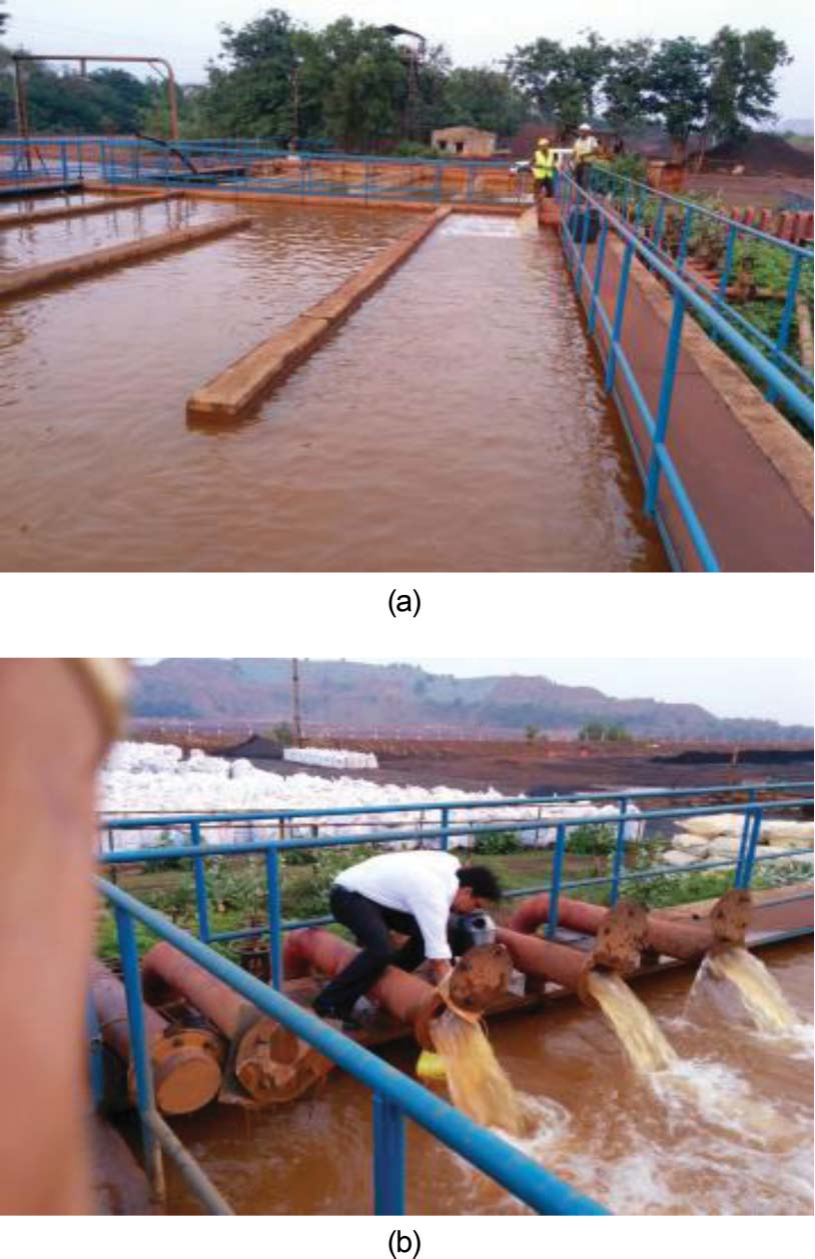
Sample collection locations at Sukinda chromite mines, Orissa, India

For the present studies, water samples were collected from quarry water storage area (tailing pond recirculating water) of beneficiation plant (Figure 1a and b). The analysis of wastewater from mines was carried out for estimation of physico-chemical parameters before and after the phytoremediation experiments.

### Analysis of SCM processed water quality

Wastewater was collected in plastic jars from SCM area and physicochemical analysis such as pH, total dissolved solids (TDS), dissolved oxygen (DO), BOD, COD, Ca, Mg, Cr(VI), SO_4_^2−^, Fe before and after the phytoremediation was performed. The value at zero (0) day was noted as initial value while the value recorded after the phytoremediation was indicated by final value. Here, zero (0) day means initial day.

### Standard experimental procedure

One plant was selected for each standard experiment. The standard procedure was carried out with known concentration of potassium dichromate (K_2_Cr_2_O_7_) (AR grade) solution. Water hyacinth plant was maintained in tap water supplemented with individual addition of 0.5, 0.75, 1, 2, and 5 mg/L chromium solution, respectively. Plastic polypropylene jar with individual addition of standard chromium solution (not using plants) was used as control. The test durations were 5, 10, and 15 days and compared with 0 day. All experiments were performed in triplicates. After specified days, water was collected from each plastic jar and filtered through Whatman no 1 filter paper. The changes of Cr (VI) concentration were measured. The maximum limit of water hyacinth to uptake hexavalent chromium at different concentration was determined.

### Experiment setup and procedure

The experiments were conducted in a series of rectangular plastic container containing 5 and 100 L of water from SCM, respectively, at an ambient temperature range (25–30°C). 250 g (for 5 L water) of fresh biomass of *Eichhornia crassipes* was cultured in a tub for the period of 7 days, whereas one of them was used as control and again the fresh biomass was noted for the following experiments. Plastic jar with only SCM effluent (not using plant) was used as control. The test durations were 0 (initial day), 5, 10, and 15 days, respectively. Deionized water was added daily to compensate the water loss through plant transpiration, sampling, and evaporation. The changes in pH, TDS, and Cr(VI) concentrations were determined at regular intervals. After the completion of each test duration, *E. crassipes* were separated into leaves, shoots, and roots for the analysis of Cr(VI) accumulation, bio-concentration factor. The TDS and DO of the treated water were also measured to know the effect of accumulation on the plant. After each experiment, plants were recycled for the next experiment. This is repeated 5–6 times. After a certain time, aquatic plants were harvested. If the plants are not harvested then after their death and decomposition, nutrients will be released back into the water system. Therefore, regular harvesting was done to avoid this process of release back of nutrients. After getting successful results, large-scale experiment carried out in 100 L chromium-contaminated processed water from SCM. For this experiment, 5 kg (for 100 L water) of fresh biomass of *E. crassipes* was used.

### Analysis of pH

pH of the mines wastewater was measured using the pH meter (Systronics, India, digital pH meter, model no: 335). The variation of pH during phytoremediation experiment with the passage of time was measured. At initial day the pH was 8.26.

### Estimation of TDS

TDS are a measure of the combined content of all inorganic and organic substances contained in a liquid in molecular, ionized or micro-granular (colloidal sol) suspended form. TDS was observed at a regular basis during phytoremediation experiment using the TDS meter (Systronics, India, model no: 308).

### Analysis of BOD and COD

The chromium-contaminated water was collected from Sukinda. The water was filtered according to the standard procedure. Then, its BOD and COD were estimated at 27°C.

### Determination of Cr (VI) concentration spectrophotometrically

Hexavalent chromium concentration in ppm was measured in spectrophotometer (Thermo scientific, Genesys 10S UV-VIS spectrophotometer). The wavelength used was 540 nm. This was performed after a purple color was developed by using diphenylcarbazide (DPC) solution according to the standard procedure (APHA 2005).

### Determination of other elemental concentration

Apart from chromium, processed water from SCM contains other elements including Ca, Mg, Fe, Si, S0_4_^2−^, etc. These elemental concentrations in the SCM water were determined before and after experiment by inductively coupled plasma atomic emission spectroscopy (ICP-AES) (Spectro Arcos). The nutrients remaining in the SCM water were measured to assess the removal potential of water hyacinth. The instrument's working wavelengths were set as suggested by the APHA (2005).

### Bio-concentration factor

The BCF provides an index of the ability of the plant to accumulate the metal with respect to the metal concentration in the substrate. The BCF was calculated as follows (Zayed *et al.* 1998). BCF is a useful parameter to evaluate the potential of the plants in accumulating metals/metalloids and this value was calculated on dry weight basis. (Lu *et al.* 2004; Yoon *et al.* 2006).
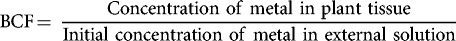



### Relative growth rate

Relative growth rate of plants were estimated after the experiment and compared with the plants' initial weight. In the standard experiment, relative growth rate was calculated according to the following equation (Hunt 1978; Bianconi *et al.* 2013).
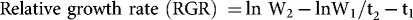
where W_1_ and W_2_ are initial fresh biomass and final fresh biomass at harvest, respectively, and (t_2_−t_1_) is the duration of the experiment in days. The results are expressed as increase of biomass per unit mass per day (gg^−1^ d^−1^).

### Extraction of metals from plant

The plants were removed from the phytoremediation tank after completion of experiment and digested according to the method of Carvalho *et al.* (2000) and Carvalho and Martin (2001). Each plant was weighed, cut, and blended. The initial fresh weight was compared with the fresh weight measured after treatment, and any physical differences observed were recorded. The plant was allowed to dry in the open air for 1 week. A dry weight of root, shoot, and stem were taken and each sample was placed in a polypropylene tube. Roots, stems, and leaves were dried in an oven at 80°C for 48 hours. The dried roots, stems, and leaves were ground into fine powder by using mortar. The powders are digested by 5 ml 1 M nitric acid (HNO_3_) and 5 mL of deionized water were added and warmed for 10–15 minutes without boiling (90–95°C). The sample was then allowed to cool. Another 5 mL of 1 M nitric acid was added and the sample was warmed for 30 minutes. Finally, 5 mL of hydrochloric acid (2 M) was added and the mixture was refluxed for 10–15 minutes. The sample was cooled to room temperature and then diluted to 100 mL with 6% (v/v) HCl. The final volume of the samples is made up to the mark in a 100-ml standard volumetric flask. From that solution, total chromium was determined by inductively coupled plasma atomic emission spectroscopy (ICP-AES) (Spectro Arcos) technique. The instrument's working wavelengths were set as suggested by the APHA (2005). Cr(VI) was analyzed using standard method according to user manual of (Perkin-Elmer 200) flame atomic spectroscopy (FAAS).

### Statistical analysis

The mean number of pH, TDS, relative growth, and concentration of Cr(VI) of processed water from SCM and synthetic water, respectively, were calculated before and after the experiment and subjected to Student's *t*-test and significant differences were calculated between treatments (Winer 1981).

## Results and discussion

In the present work, studies on the removal of Cr(VI) were carried out by phytoremediation technique using water floating macrophytes *E. crassipes*. The technique used in this process is called more appropriately rhizofiltration technique, which is a part of phytoremediation. The phytoremediation studies were performed as a function of relative growth, BCF, TDS, accumulation, and toxicity. Experiment was carried out initially in 5 L of water and then large-scale experiment was done in 100 L of SCM water. The quality of the processed water from SCM was determined before and after the treatment with water hyacinth plants. The results for all the parameters determined are presented in Table 1. For standardization, the experiment was first carried out by adding known concentration of 0.5, 0.75, 1, 2, 5 mg/L chromium solution, respectively. The results indicate that the water hyacinth is very efficient in reducing the Cr(VI) concentration (Figure 4) and also hardness in mines wastewater (Table 1) in 5 L as well as in 100 L of processed water from SCM.

**Table 1.** Characteristics of elements of processed water from Sukinda chromite mines before and after the phytoremediation experiment.

Parameters	Initial (0 Day) value Conc (ppm) Mean ± SE	Final (15 Day) Conc (ppm) Mean ± SE
Ca	16 ± 0.45	10 ± 0.45^*^
Mg	46.23 ± 0.07	32 ± 0.45
Fe	<0.001	<0.001
SiO_2_	30 ± 0.45	24 ± 0.45^***^
SO_4_^2−^	1.7 ± 0.45	1.4 ± 0.45
Co	<0.001	<0.001
Total Cr	2.0 ± 0.071	0.01 ± 0.005^***^
Cr(VI)	2.0 ± 0.071	0.01 ± 0.005^***^
Ni	<0.001	<0.001
Ti	<0.001	<0.001
TDS	290 ± 0.0089	235 ± 1.11^***^

SE, standard error. Data represents mean ± SE of n = 5,

* p < 0.05,

*** p < 0.001 (Student's *t*-test).

### pH

**Figure 2.**
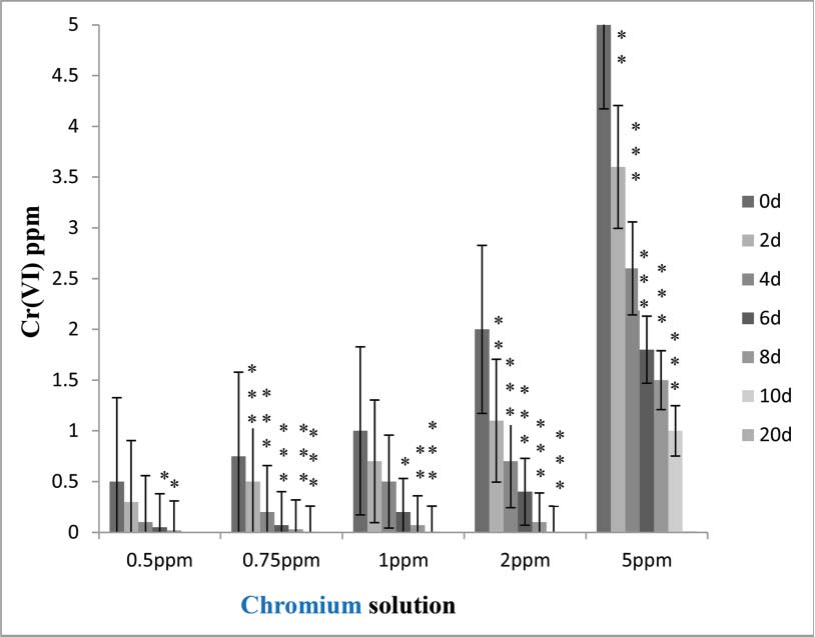
Effect of time period on the absorption of chromium from standard solution by water hyacinth. Data represents mean ± SE of n = 5 *p < 0.05, **p < 0.01***p < 0.001 (Student's *t*-test).

After collection of processed water from SCM, pH was estimated as 8.23. The results before and after the biological treatment with water hyacinth showed a reduction in the pH of the wastewater (Figure 2). In case of control, there was no change of pH. The pH was reduced to nearly neutral in all cases studied. It can be interpreted that the reduction in pH might be due to absorption of pollutants by plant. The reduction in pH favored microbial action to degrade BOD and COD in the wastewater (Mahmood *et al.* 2005). The reason behind the decrease of pH of water is given below. At pH 6.5–8.5 the overall oxidation reaction is
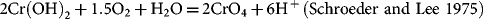



Given the standard free energy change for this equation (Latimer 1952) as +12.7 Kcal mol^−1^, at pH = 7.0 and P_02_ = 0.21 atm, at equilibrium
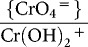
which indicates that under oxygenated natural water conditions Cr(VI) is the thermodynamically stable species.

### TDS

The TDS of the processed water from SCM was estimated and the reading was noted in parts per million (ppm) (Table 1). During lab study, it was observed that TDS of processed water from SCM was gradually decreased with the passage of time due to removal of Cr(VI) from water (Figure 4). There was no alteration of TDS in case of control sample. On subsequent days, it was observed that the TDS value considerably decreased by the accumulation process. During phytoremediation, *E. crassipes* has the capacity to reduce hardness of water by the removal of Ca and Mg at a certain amount (Table 1).

### Elemental concentration of standard and processed water from SCM during phytoremediation

Five different chromium concentrations, *i.e.*, 0.5, 0.75, 1, 2, 5 mg/L, were used in different experimental sets. The variations of the metal concentrations with increasing time span in the experimental sets are shown in Figure 2.

**Figure 3.**
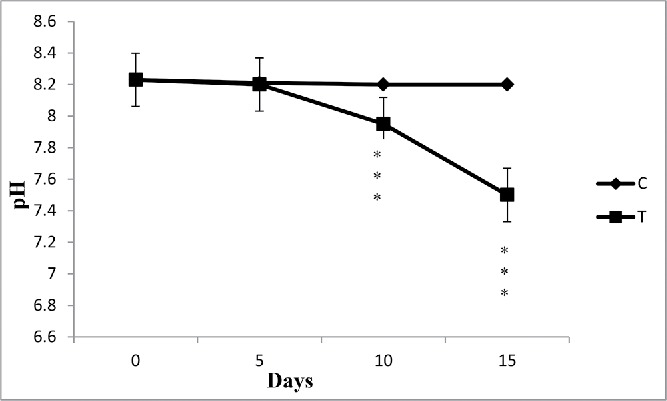
The effect of water hyacinth treatment on pH. In this figure, C stands for control and T stands for treatments. Data represents mean ± SE of n = 5 ***p < 0.001 (Student's *t*-test).

Results revealed increasing trend of removal with the increasing incubation period. With increasing metal concentration water hyacinth was able to remove and accumulate decrease amount of heavy metals. In case of treatments, control experimental sets showed loss of 2.1–5.7% heavy metals from the water (Figure 5). This loss might be due to precipitation, adsorption to clay particles and organic matter, and co-precipitation with secondary minerals. Results showed significantly higher (p < 0.001) accumulation of Cr (VI) in *E. crassipes* (Figure 5). Higher removal of Cr (VI) may be attributed to the several special characters of *E. crassipes* like fibrous and dense root system, broad leaves, and fast growth (Mant *et al.* 2007). Attempt has been made in this work to estimate chromium (VI) absorption from standard as well as wastewater from mines using *E. crassipes* to develop the phytoremediation potential of this plant. The results showed that initially, Cr (VI) levels in mine wastewater were 2.0 mg/L. During phytoremediation, the reduction of Cr (VI) concentration in mine wastewater (5 L: Figure 5 as we as in 100 L: Figure 6a and b) with the passage of time indicated a change from toxic (2 mg/L) to nontoxic (0.01 mg/L) form. The data revealed that a significant amount (99.5%) of hexavalent chromium was removed from the water (Figure 5). The water hyacinth appeared to be a good choice for removing Cr(VI) from polluted water. Research has shown that water hyacinth is especially adept at removal of Cr(VI) from wastewater (Mohanty and Patra 2011; Mohanty *et al.* 2012). At low standard chromium concentrations (0.25–2 ppm), the plant removed about 90% of the chromium in the water. As the concentrations increased (at 5 mg/L), the plant appeared to be able to take up as much percent of Cr (VI) (significant amount), but more time (29 days) was required to take up hexachrome from water. It was reported that the water hyacinths can phytoremediate metals such as potassium, iron, magnesium, and calcium. Water hyacinth has been reported to bioaccumulate some of these metals (Carbonell *et al.* 1998; Zhu *et al.* 1999; Ingole and Bhole 2003; Mahmood *et al.* 2005; El-Gendy *et al.* 2006; Tiwari *et al.* 2007; Upadhyay and Tripathi 2007). The concentration of dissolved elements other than hexavalent chromium (calcium, magnesium, sulfate, etc.) that remained in the processed water from SCM after phytoremediation is shown in Table 1. After phytoremediation, hardness of mines wastewater also reduced. Studies have shown that the phytoremediation efficiency of metals greatly depends on the concentration of such metals in solution, and the higher the concentration of the metals in the solution the lower the removal efficiency (Carvalho and Martin 2001; Ingole and Bhole 2003; Keith *et al.* 2006). It has not been long since water hyacinth was used commercially as a wastewater cleaning source. It has been recognized as very useful in domestic wastewater treatment also (Dinges 1976; Wolverton and McDonald 1979; Valipour *et al.* 2015).

**Figure 4.**
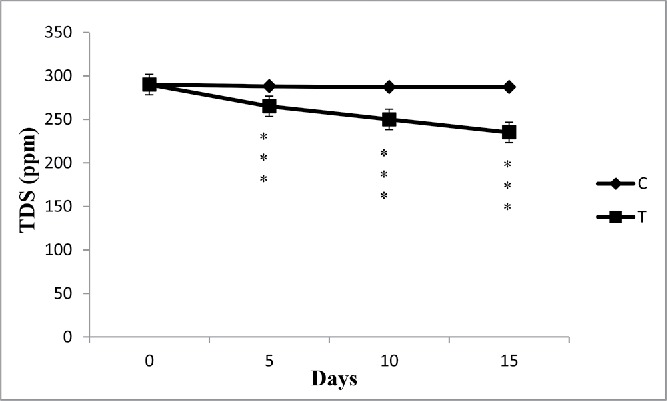
The effect of water hyacinth treatment on TDS. In this figure, C stands for control and T stands for treatments. Data represents mean ± SE of n = 5 ***p < 0.001 (Student's *t*-test).

**Figure 5.**
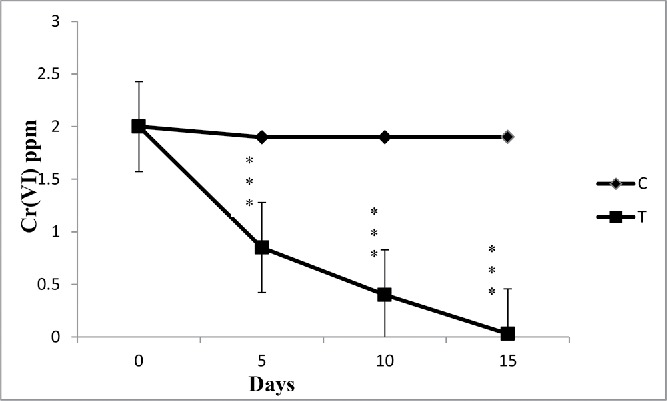
The effect of water hyacinth treatment on chromium removal (in 5 L SCM water). In this figure, C stands for control and T stands for treatments. Data represents mean ± SE of n = 5 ***p < 0.001 (Student's *t*-test).

**Figure 6.**
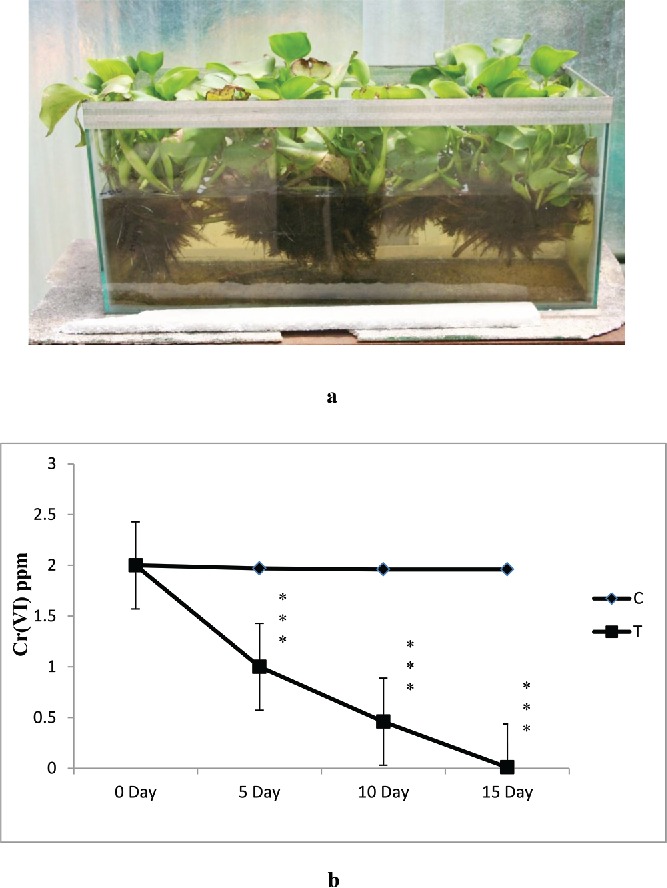
(a) Phytoremediation experiment in 100 L SCM processed water. (b) The effect of water hyacinth treatment on chromium removal (in 100 L SCM water). In this figure, C stands for control and T stands for treatments. Data represents mean ± SE of n = 5 ***p < 0.001 (Student's *t*-test).

### Permeable limits of water hyacinth to uptake hexavalent chromium

In the experiment, first stock solution of chromium was prepared at different concentrations to determine the permeable limit of water hyacinth to uptake hexachrome. The results indicated that water hyacinth effectively removed chromium from 0.5 to 2.0 ppm stock solution, respectively. From standard experiment data, it was observed that, at lower concentration (0.5), water hyacinth is very efficient in reducing 99% of Cr (VI) from water. Also, in case of 0.5 ppm standard chromium solution, minimum time was (only 8 days) required to remove 99% hexachrome from water. In addition to that, in case of other standard solutions (0.75–2 mg/L) maximum 10–15 days were required to remove chromium as compared to 20 days required at higher concentration (5 mg/L) (Figure 2). According to the United States Environmental Protection Agency (USEPA), the maximum permissible limit for Cr(VI) for discharge into inland surface waters is 0.1 mg/L and in potable water is 0.05 mg/L (EPA 1999).

### Reduction in BOD and COD

In general, all the waste samples collected were devoid of dissolved oxygen (DO). After phytoremediation experiment, there was increase in the DO after treatment as indicated by reduction of BOD and COD in the wastewater. In present study, the introduction of water hyacinth in SCM wastewater has shown 50% and 34% reduction in BOD and COD, respectively (Table 2). The reduction in BOD and COD can result in an increase in DO concentration of wastewater. According to Reddy (1981), the presence of plants in wastewater decrease dissolved CO_2_ during the period of high photosynthetic activity. This photosynthetic activity increases the DO of water, thus creating aerobic conditions in wastewater which favor the aerobic bacterial activity to reduce the BOD and COD. Tripathi and Shukla (1991) reported 96.9% reduction in BOD using *Eichhornia crassipes* and algae for sewage wastewater. The decrease in COD and BOD can result in a 67% increase in DO concentration of wastewater. The use of water hyacinth as the functional unit in wastewater treatment systems was increasingly examined and treatment regimens developed after successful pilot projects (Brix and Schierup 1989; Mandi 1994; Sinha *et al.* 2014). Wastewater treatment with water hyacinth was successfully implemented in the city of San Diego, USA, to develop a treated effluent attaining quality standards that would be expected from advanced secondary treatment processes (Tchobanoglous *et al.* 1989).

**Table 2.** Characteristics of processed water from Sukinda chromite mines before and after the phytoremediation experiment.

SCM water	pH Mean ± SE	BOD (mg/L) 27°C-3 days Mean ± SE	COD (mg/L) 27°C-3 days Mean ± SE
0 day	8.26 ± 0.0089	352 ± 0.45	423 ± 0.45
15 day	7.5 ± 0.089	176 ± 0.45^***^	279 ± 0.45^***^

SE, standard error. Data represents mean ± SE of n = 5,

*** p < 0.001 (Student's *t*-test).

### Effect of bio-concentration factor

Bio-concentration factor (BCF) is a useful parameter to evaluate the potential of the plants in accumulating metals/metalloids and it was calculated on dry weight basis. The ambient metal/metalloid concentration in water was the major factor influencing the metal/metalloid uptake efficiency. In general, when the metal/metalloid concentration in water increases, the amount of metal/metalloid accumulation in plants increases, then the BCF decreases (Wang and Lewis 1997; Karimi *et al.* 2009). BCF is a common important factor when considering the phytoremediation potential of a given species (Zhao *et al.* 2003). In this study, BCF of chromium of SCM water indicated the phytoremediation potential for this toxic metal. The BCF of chromium significantly increased with the passage of time and maximum factor showed at 15 days (Table 3). BCF of Cr was maximum at the highest growing period of water hyacinth plants (with high biomass). In plants treated with different chromium concentrations, the bio-concentration factor at final day (day 15) increased in all treatments (0.5, 0.75, 1.0, 2.0, 5.00 mg/L), while in 5.0 ppm standard solution, final BCF was low as compared to other treatments (Table 4). The maximum BCF (0.98) for Cr was found in both 0.5 and 1.0 mg/L of chromium solution on day 15, indicating that *E. crassipes* can be used for effective phytoremediation. Zhu *et al.* (1999) examined that the BCF of *E. crassipes* are very high for Cd, Cu, Cr, and Se at low external concentration, and they are found to decrease with the increase in external concentration (Mohanty *et al.* 2012). Cr accumulation and bio-concentration factor generally increased with age of the plants as well as with increase in external Cr availability in water. It represents a cost-effective, eco-friendly plant-based technology for the removal of metals from the environment and has great potential for future applications.

**Table 3.** BCF of chromium of processed water from Sukinda chromite mines.

SCM water	5day	10day	15day
Chromium (VI)	0.3 ± 0.004	0.55 ± 0.0089^***^	0.85 ± 0.0089^***^

SE, standard error. Data represents mean ± SE of n = 5,

*** p < 0.001 (Student's *t*-test).

**Table 4.** The effects of chromium on bio-concentration factor (BCF) of *Eichhornia crassipes* at different concentration and exposure time.

Standard chromium solution (ppm)	5 day	10 day	15 day
0.5	0.2 ± 0.045	0.5 ± 0.045	0.98 ± 0.006^**^
0.75	0.53 ± 0.004	0.7 ± 0.045	0.97 ± 0.006^***^
1.0	0.45 ± 0.004	0.7 ± 0.045	0.98 ± 0.004^***^
2.0	0.39 ± 0.006	0.67 ± 0.004	0.92 ± 0.006^***^
5.0	0.2 ± 0.045	0.45 ± 0.004	0.67 ± 0.004^*^

SE, standard error. Data represents mean ± SE of n = 5,

* p < 0.05,

** p < 0.01,

*** p < 0.001 (Student's *t*-test).

### Relative growth rate

**Figure 7.**
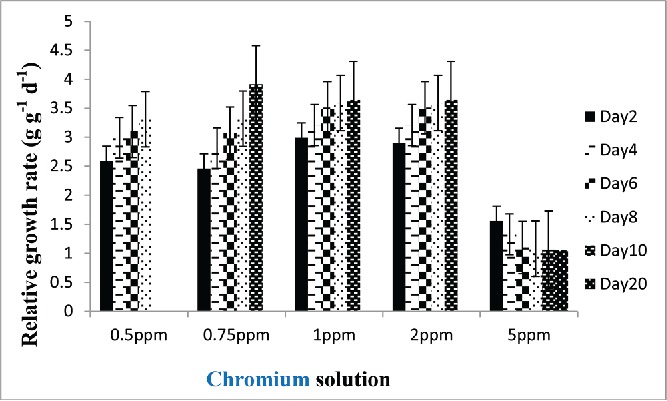
The effect of different standard chromium concentration on relative growth of aquatic plant.

Relative growth is considered to be the most widely used method for estimating plant growth. In the present study, the effects of different concentrations of chromium solution on relative growth of *E. crassipes* were studied and the results are presented in Figure 7. As indicated from data in plants treated with different strengths of chromium, the relative growth increased with 0.50–2 mg/L of chromium but decreased with 5 mg/L of chromium (Figure 7). It appears that low concentration of hexavalent chromium could stimulate plant's growth. In plants, Cr (VI) at low concentrations (0.05–2 mg/L) was found to promote growth and increase yield, but it is not considered essential to plants (Peralta-Videa *et al.* 2009; Paiva *et al.* 2009). While studying the uptake of some heavy metals by water hyacinth, Ingole and Bhole (2003) reported that at lower concentrations of heavy metals, the plant growth was normal and removal efficiency was greater. At higher concentrations (5 mg/L), the plant started wilting and removal efficiency was reduced to 39% as compared to the initial concentration. Zaranyika and Ndapwadza (1995) were observed that Cr (VI) tolerance and accumulation in selected *E. crassipes* growth are mainly by suppressing development of new roots and reducing relative growth rates to about 15% of those controls. Several researchers have reported similar results (Gupta *et al.* 2009; Murányi and Ködöböcz 2008). The best way of long-term strategy for improving phytoaccumulation is to understand and exploit the biological processes involved in metal/metalloid acquisition, transport, and shoot accumulation. This is due to higher concentrations of heavy metal/metalloid ions in solution and also their inhibitory effects on plant metabolic activity, alternatively reduced growth of plants, leaf necrosis, and inhibited plant physiology systems.

### Chromium accumulation and translocation

**Figure 8.**
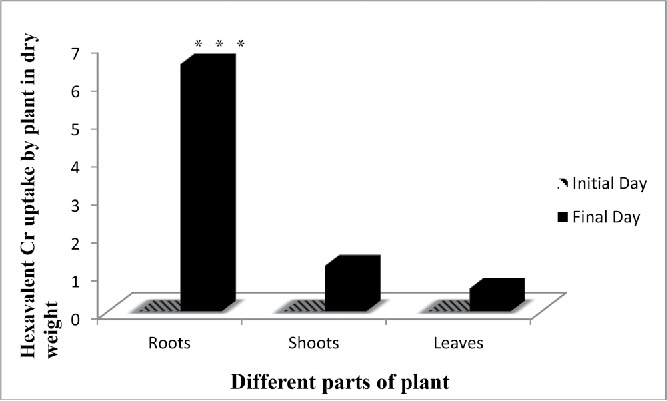
Accumulation of Cr (VI) by aquatic plant. Data represents mean ± SE (Standard error) of n = 5 ***p < 0.001 (Student's *t*-test).

**Figure 9.**
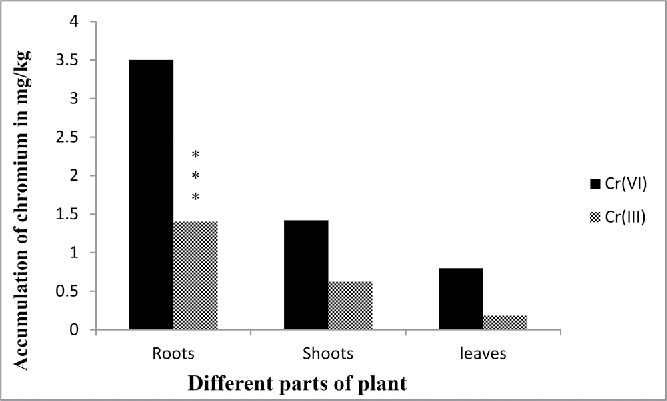
Different forms of chromium after accumulation by different parts of plants. Data represents mean ± SE (Standard error) of n = 5 ***p < 0.001 (Student's *t*-test).

The bioaccumulation pattern of Cr(VI) in leaves, stems, and roots of *E. crassipes* showed that the roots have the highest concentration followed by leaves and stems, respectively. Around 50–60% of total Cr accumulation in the plants was present in the roots. While, not all chromium are in hexavalent form, 20% is in trivalent form that is in roots Cr(VI) accumulated 3.5 mg/kg and Cr(III) was 1.4 mg/kg (Figures 8 and 9). Previous studies on the accumulation of various metal ions by aquatic plants have also shown that the deposition of most metals was higher in roots than the other parts of plants (Hossner 1996). Movement of metal/metalloids containing sap from the root to the shoot, termed translocation, is primarily controlled by two processes root pressure and leaf transpiration. Some metals are accumulated in roots, probably due to some physiological barriers against metal/metalloid transport to the aerial parts (Rahman *et al.* 2008; Bose *et al.* 2008). As shown in the results, total chromium content in roots and shoots showed significant differences in its distribution and also in the distribution of Cr(III) and Cr (VI) content (Figure 9). Cr accumulation was more in roots than shoots during all stages of plant growth. Maximum accumulation of total Cr was observed in roots. It is observed by several researchers that Cr accumulates mainly in roots and shoots; however, roots accumulate the major part, with only a small part translocated to the shoots (Sundaramoorthy 2010; Paiva 2009). Accumulation of Cr in the different parts of the plant was in the following order roots > stem > leaves > seed (Tiwari *et al.* 2009). Corroborating these results are the findings of several works; for instance, Huffman and Allaway (1973) found that bean seeds accumulated about 0.1% Cr, while roots accumulated 98% Cr. Furthermore, Liu and co-workers, (2008) studied hydroponically grown *A. viridis* L. under different concentrations of Cr (VI) and found that Cr was accumulated primarily in roots (Liu *et al.* 2008). Another study performed by Vernay *et al.* (2007) in *Lolium perenne* grown in the presence of 500 μM of Cr (VI) showed that roots accumulated 10 times more Cr than leaves. Spinach (*Spinacia oleracea* L. cv. “Banarasi”) grown in the presence of Cr (VI) showed more accumulation of Cr in the roots than in leaves and the stem showed the least accumulation (Gopal *et al.* 2009). Also, in celery seedlings grown in the presence of Cr(III), most Cr was accumulated in roots (Scoccianti *et al.* 2006). Higher Cr bioaccumulation in roots in comparison to shoots was also reported earlier by several researchers (Katz and Salem 1994; Mishra *et al*. (1995); Zayed *et al.* 2003; Pulford and Watson 2003; Ghosh and Singh 2005; Dong *et al.* 2007; Zhang *et al.* 2007). High bioavailability of Cr in roots and its low translocation to shoots is a common phenomenon (Dickinson and Lepp 1997); High Cr accumulation in root cells was supported by Shanker *et al.* (2004), who suggested immobilization of chromium from the vacuoles. Metalloid ions penetrated plants by passive process, mostly by exchange of cations which occurred in the cell wall. All heavy metals were taken up by plants through absorption and translocation and released by excretion. It can be proposed that the roots reached saturation during the period and there exists some mechanism in roots that could detoxify heavy metals or transfer them to aerial parts.

### Reuse of water hyacinth plant after experiment

According to Lindsey and Hirt (1999), water hyacinth can be used as food for people or fodder because its leaves are rich in proteins and vitamin A. However, it is not recommended for consumption if used for removal of heavy metals and toxic substances as it can cause problems when entering into the food chain (Chua 1998). Its biomass is rich in nitrogen and other essential nutrients. Apart from biogas (Singhal and Rai 2003), its sludge contains almost all nutrients and can be used as a good fertilizer with no detrimental effects on the environment (Patil *et al.* 2011). After harvesting, it can be used for composting, anaerobic digestion for production of methane, and fermentation of sugars into alcohol (Patil *et al.* 2011), green fertilizer, compost, and ash in regenerating degraded soils. These operations can help in recovering expenses of wastewater treatment.

## Conclusion

The water hyacinth was found to be efficient in reducing the concentrations of TDS, pH, Cr(VI) concentration of SCM water within 15 days of treatment. The experimental results showed that this plant has performed extremely well in removing 99.5% Cr(VI) from processed water of SCM during 15 days of experimental period. Removal efficiency (99.5%) was same in 5 L and also in 100 L of processed water from SCM and retention period is also the same (15 days). This aquatic plant was also effective in reduction of BOD and COD. At this time of water, energy, and environmental purity crisis, water hyacinth can be a very effective tool in cleaning the wastewater after bio filtration treatment. The novelty of this work is that it was not only a laboratory-scale baseline study, but also a large-scale experiment in 100 L of processed water from SCM. Earlier researchers have done many research on chromium removal from different wastewater, while large-scale experiment was never done performed before in mines water. Our future aim will be to develop pilot plant in SCM, Orissa, India, for implementation of this work for industrial purposes. It is hoped that in future more attention will be paid to water hyacinth for treatment of industrial as well as other kinds of wastewaters.
